# Qili Qiangxin ameliorates chronic heart failure: a randomized clinical trial of biomarkers, inflammation, and cardiac outcomes

**DOI:** 10.3389/fphar.2025.1605944

**Published:** 2025-09-30

**Authors:** Feng Zhu, Rui Hu, Chao Lv, Jin Wang, Xuqin Du, Xudong Zeng, Yuxuan Huang, Yiming Ma, Cheng Yang, Fengjie Guo

**Affiliations:** ^1^ Chongqing University of Chinese Medicine, Chongqing, China; ^2^ Chongqing Academy of Chinese Materia Medica, Chongqing, China; ^3^ Internal Medicine of Traditional Chinese Medicine, Chongqing University Three Gorges Hospital, Chongqing, China; ^4^ Department of Cardiovascular Disease, ZiBo Hospital of Traditional Chinese Medicine, Zibo, China; ^5^ The South China University of Technology School of Medicine, Guangzhou, China

**Keywords:** chronic heart failure, Qili Qiangxin, trimethylamine N-oxide, intestinal permeability, D-lactate, intestinal fatty acid–binding protein

## Abstract

**Objective:**

To evaluate the effects of the Chinese botanical formulation Qili Qiangxin (QLQX) on clinical outcomes, cardiac function, systemic inflammation, and intestinal permeability in patients with chronic heart failure (CHF).

**Methods:**

In a randomized, controlled, parallel-group trial, 110 patients with CHF were assigned to receive QLQX plus standard-of-care (n = 55) or standard-of-care alone (n = 55); 101 participants completed follow-up. Clinical assessments included New York Heart Association (NYHA) functional classification, Minnesota Living with Heart Failure Questionnaire (MLHFQ) scores, and echocardiography. Serum B-type natriuretic peptide (BNP), tumor necrosis factor-alpha (TNF-α), interleukin-6 (IL-6), trimethylamine-N-oxide (TMAO), D-lactate (D-LA), and intestinal fatty acid-binding protein (I-FABP) were measured to assess systemic inflammation and intestinal barrier injury. Correlation between biomarker levels and CHF severity indices were examined.

**Results:**

Compared with control, the QLQX exhibited significant improvements in NYHA class (p < 0.05) and MLHFQ scores. The QLQX group showed significant reductions in BNP, TNF-α, IL-6, and TMAO (p < 0.01), indicating attenuation of systemic inflammation. D-LA and I-FABP were significantly lower with QLQX (p < 0.05), suggesting improved intestinal barrier integrity. Higher levels of TMAO, TNF-α, IL-6, D-LA, and I-FABP correlated with worse NYHA class and lower left ventricular ejection fraction (LVEF) (p < 0.05), supporting their association with CHF severity.

**Conclusion:**

As an adjunct to standard therapy, QLQX improved clinical status and quality of life in CHF and favorably modified circulating biomarkers of cardiac stress, systemic inflammation, and intestinal barrier injury. These findings support a link between systemic inflammation, gut barrier dysfunction, and CHF severity. Larger, multicenter trials with mechanistic evaluations are needed to confirm efficacy and clarify QLQX’s molecular targets.

## Introduction

Chronic heart failure (CHF) is a clinical syndrome characterized by impaired cardiac function and reduced cardiac output, leading to dyspnea, fatigue, exercise intolerance, and diminished quality-of-life (Heidenreich et al., 2022). With rising global prevalence, CHF imposes substantial morbidity, mortality, and economic burden (Ribeiro et al., 2022). Although guideline-directed medical therapy improves survival and symptoms, residual risk remains high, indicating the need for adjunctive strategies that target pathophysiological domains insufficiently addressed by current therapies (Zick et al., 2005).

Systemic inflammation, endothelial dysfunction, and metabolic disarray are prominent in CHF and contribute to adverse remodeling and progression (Halade et al., 2022; Güder et al., 2007). Increasing evidence implicates the gut–heart axis in CHF: reduced intestinal perfusion and congestion can compromise mucosal integrity, while dysbiosis alters the production of microbially derived metabolites that access the circulation and may exacerbate inflammation and cardiometabolic stress (Sun et al., 2022; Paraskevaidis et al., 2024). TMAO, formed by hepatic oxidation of trimethylamine generated by gut microbiota from dietary precursors such as choline and carnitine, has been associated with incident cardiovascular events and with worse outcomes in heart failure (Konieczny and Kuliczkowski, 2022). Circulating D-lactate (a bacterial fermentation product) and intestinal fatty acid–binding protein (I-FABP, released with enterocyte injury) are informative markers of intestinal barrier injury; elevated levels are consistent with increased barrier disruption and translocation risk (Rivera et al., 2025).

Qili Qiangxin (QLQX) is a standardized, proprietary Chinese botanical drug formulation authorized by the China National Medical Products Administration for CHF. Preclinical and clinical studies suggest anti-inflammatory, antifibrotic, and cardioprotective activities and improvements in symptoms and natriuretic peptides when used as adjunctive therapy (Li et al., 2013; Cheang et al., 2024; Wu et al., 2025). However, effects on key inflammatory mediators (for example, TNF-α and IL-6), microbiota-dependent metabolites (for example, TMAO), and circulating markers of intestinal barrier injury (for example, D-lactate and I-FABP) remain insufficiently characterized in randomized clinical settings (Xiang et al., 2022; Wei et al., 2022).

This randomized controlled trial evaluated the effects of QLQX added to standard-of-care on clinical status (NYHA class and MLHFQ), echocardiographic parameters, and circulating biomarkers of cardiac stress, systemic inflammation, and intestinal barrier injury in adults with CHF. We further explored associations between biomarker levels and CHF severity indices, including NYHA class and LVEF, to contextualize potential mechanistic links. The overarching objective was to clarify the therapeutic potential of QLQX within the gut–heart–inflammation framework and to inform the design of larger mechanistic studies.

## Methods

### Study design and setting

This was a prospective, randomized, controlled, parallel-group trial conducted in the Department of Cardiovascular Medicine, Zibo Traditional Chinese Medicine Hospital, Shandong Province, China, from October 2020 to February 2022. Patients with chronic heart failure (NYHA II-IV) were enrolled and randomized 1:1 to receive guideline-directed medical therapy (GDMT) alone (control) or GDMT plus Qili Qiangxin (QLQX) capsules for 8 weeks.

The study followed the Declaration of Helsinki and applicable Chinese regulations. The protocol was approved by the Ethics Committee of Zibo Traditional Chinese Medicine Hospital (Approval No. 2021-ethics-004). Written informed consent was obtained from all participants or their legally authorized representatives before any study procedures. Sonographers performing echocardiography and the statistician were blinded to group allocation; participants and treating physicians were not blinded (PROBE design: prospective randomized open trial with blinded endpoint evaluation).

Investigational product: composition, quality control, and regulatory status QLQX is an approved proprietary traditional Chinese medicine officially authorized by the China NMPA for the treatment of chronic heart failure (Approval Number: Z20040141). QLQX capsules were provided by Shijiazhuang Yiling Pharmaceutical Co., Ltd. (Shijiazhuang, China). The formulation is standardized and consists of pharmacopeial-grade botanical drugs as specified in the Chinese Pharmacopoeia and the manufacturer’s regulatory dossier. Exact quantities and botanical drug details are provided in Supplementary Table S1.

### Diagnostic criteria

Chronic heart failure was diagnosed by two independent cardiologists according to the 2018 Chinese Guidelines for the Diagnosis and Treatment of Heart Failure (Chinese Medical Association, Cardiovascular Branch), in conjunction with the Framingham diagnostic criteria. The diagnostic framework included.

• Symptoms: exertional dyspnea, orthopnea, paroxysmal nocturnal dyspnea, fatigue, reduced exercise tolerance, dependent edema, cough productive of frothy sputum, and palpitations.
• Objective findings: physical examination and corroborating evidence from chest radiography, echocardiography, and electrocardiography.
• Biomarker confirmation: BNP ≥35 pg/mL.

### NYHA functional classification

Functional status was assessed and graded by two independent cardiologists using the New York Heart Association (NYHA) classification.

• Class I: no limitations of physical activity; ordinary physical activity does not cause undue fatigue, palpitation or shortness of breath.
• Class II: slight limitation of physical activity; comfortable at rest; ordinary physical activity results in fatigue, palpitation, shortness of breath or chest pain.
• Class III: marked limitation of physical activity; comfortable at rest; less than ordinary activity causes symptoms.
• Class IV: symptoms at rest; any physical activity causes discomfort.

### Eligibility criteria

#### Inclusion criteria

• CHF (NYHA II-IV) confirmed by at least two attending physicians.
• Age 45–82 years.
• Disease duration ≥3 months.
• Written informed consent from patients or a legally authorized representative.

#### Exclusion criteria

• Severe dysfunction of vital organs, primary hepatic, renal, endocrine, or hematopoietic diseases.
• Mental illness or poor treatment adherence anticipated by the investigator.
• Severe, unstable heart failure requiring advanced support (e.g., inotropes, mechanical ventilation), per investigator judgment.
• Pregnancy or lactation.
• Use of probiotics or antibiotics within 4 weeks prior to enrollment.
• Chronic or acute diarrhea.
• Evidence of active infection within the previous week.

### Randomization and treatment protocol

A computer-generated random sequence prepared by an independent statistician. Allocation concealment used sequentially numbered, opaque, and sealed envelopes opened in sequence after enrollment. Echocardiography were performed by sonographers blinded to treatment assignment. The trial used an open-label design for participants and treating clinicians, with blinded endpoint assessment where feasible (echocardiography).

### Interventions

• Control Group: GDMT per treating physician, which could include diuretics, nitrates, digoxin, angiotensin-converting enzyme inhibitors or angiotensin receptor blockers, and beta-blockers, administered for 8 weeks.
• QLQX Group: the same GDMT plus Qili Qiangxin capsules, four capsules per dose, three times daily, for 8 weeks. Investigators could adjust dose or temporarily interrupt QLQX for adverse events.

Safety monitoring Adverse events were recorded throughout the study and managed per clinical judgment. Serious adverse events were reported to the institutional ethics committee according to local regulations.

### Outcomes and assessments

• Baseline characteristics: age, gender, height, weight, body mass index (BMI), personal history, NYHA Classification, and comorbidities.
• Efficacy and mechanistic endpoints:
  1. NYHA class change from baseline to week 8, adjudicated by two cardiologists.
  2. Echocardiography: left atrial diameter (LAD), left ventricular end-diastolic diameter (LVEDD), interventricular septal thickness (IVST), left ventricular posterior wall thickness (LVPWT), and left ventricular ejection fraction (LVEF). Heart failure phenotypes were categorized by LVEF as HFrEF (≤40%), HFmrEF (41%–49%), and HFpEF (≥50%).
  3. Imaging procedures: Two blinded sonographers used a Vivid E95 system (GE Healthcare) with a 3.5-MHz transducer. LVEF was calculated offline using the biplane Simpson’s method from apical four- and two-chamber views as the average of three consecutive cardiac cycles.
  4. Quality of life: Minnesota Living with Heart Failure Questionnaire (MLHFQ) total score (range 0–105; higher scores indicate worse quality of life).
  5. Biomarkers: Serum BNP, trimethylamine N-oxide (TMAO), intestinal fatty acid–binding protein (I-FABP), D-lactate (D-LA), tumor necrosis factor-α (TNF-α), and interleukin-6 (IL-6).

### HPLC-MS/MS assay for TMAO

Serum TMAO were quantified using high-performance liquid chromatography tandem mass spectrometry (HPLC-MS/MS). Briefly, 100 μL of thawed serum was mixed with 300 μL of LC–MS grade methanol for protein precipitation, vortexed for 2 min, and centrifuged at 16,000 g for 10 min at 4 °C. The supernatant was transferred to autosampler vials for analysis. Chromatographic separation used a Thermo Scientific Hypersil GOLD column (100 mm × 2.1 mm, 1.9 μm) at 40 °C. Mass spectrometric detection employed positive electrospray ionization. Quantification used a stable isotope–labeled internal standard (d9-TMAO) and a 7-point calibration curve.

### Assays for BNP, I-FABP, D-LA, TNF-α and IL-6

Serum BNP (abs551414, Absin), intestinal fatty acid–binding protein (I-FABP; RAB0537, Sigma), D-lactate (abs580272, Absin), tumor necrosis factor-alpha (TNF-α; abs510006, Absin), and interleukin-6 (IL-6; abs510003, Absin) were measured using ELISA kits or an enzymatic microplate assay, according to manufacturers’ instructions.

ELISA workflow: All reagents and samples were equilibrated to room temperature (approximately 30 min). Standards or appropriately diluted samples (100 μL) were added to antibody-coated wells and incubated for 90 min at 37 °C. After washing, a biotinylated detection antibody was added for 60 min at 37 °C, followed by HRP-conjugated streptavidin for 30 min. TMB substrate was added for 15 min in the dark, and the reaction was stopped. Optical density was read at 450 nm on a microplate reader (BioTek Instruments, Winooski, VT, United States). All samples were analyzed in duplicate; mean values were used for analysis.

Enzymatic microplate assay workflow: Reaction buffer (60 μL) was added to each well, followed by 20 μL of sample, standard, or distilled water (blank), and then 10 μL of enzyme and 10 μL of coenzyme solutions. After mixing and a 5-minute incubation at 25 °C, dye reagents were added, mixed, and incubated for 20 min at 25 °C in the dark. Absorbance was measured at 450 nm with blank correction.

### Statistical analysis

Statistical analyses were performed using SPSS 22.0. Data are presented as mean ± standard deviation unless otherwise specified. Descriptive statistics summarized baseline characteristics. Between-group comparisons used independent t-tests or Mann-Whitney U tests, as appropriate; within-group pre-post comparisons used paired t-tests. Categorical variables were analyzed with chi-square or Fisher’s exact tests. Correlation analyses were selected based on data distribution. Spearman’s rank correlation was used for non-normally distributed biomarkers (D-LA, I-FABP, TMAO, TNF-α, IL-6). Pearson’s correlation coefficient assessed linear relationships between circulating mediators (TMAO, TNF-α, IL-6) and normally distributed clinical parameters (MLHFQ scores, BNP levels, LVEF). To adjust for potential confounders, multivariable linear regression models included the following covariates: age (continuous), sex (categorical), BMI (continuous), and major comorbidities (hypertension, diabetes, hyperlipidemia, and history of stroke; binary variables). A two-sided p value < 0.05 was considered statistically significant.

## Results

### Clinical characteristics

A total of 110 patients with chronic heart failure were randominzed in equal numbers to control (n = 55) or Qili Qiangxin (QLQX) (n = 55). Nine patients withdrew (control: n = 5; QLQX: n = 4), yielding an overall dropout rate of 8.18%. In total, 101 participants completed the 8-week treatment period (control: n = 50; QLQX: n = 51).

Baseline demographic and clinical characteristics were well balanced between groups (Table 1). Sex distribution was similar (control: 52.0% female, 48.0% male; QLQX: 54.9% female, 45.1% male), as were age (control: 72.70 ± 5.25 years; QLQX: 71.47 ± 4.64 years), height, weight, blood pressure, heart rate, smoking and alcohol use, and BMI. All participants met eligibility criteria, including the absence of known liver or kidney disease, malignancy, autoimmune disease, inflammatory bowel disease, or irritable bowel syndrome. The mean duration of heart failure was 4.04 ± 2.14 years (control) and 3.73 ± 2.10 years (QLQX). Baseline NYHA class distribution was comparable (class I: 12.9%; class II: 68.3%; class III: 18.8%; no class IV). At baseline, heart failure phenotypes were HFrEF 11.3%, HFmrEF 33.4%, and HFpEF 55.3% (data not shown).

**TABLE 1:** Characteristics of two groups of patients.

Item	Category	Control group (n = 50)	QLQX group (n = 51)	*P*
Sex [n (%)]				0.770
	Female	26 (52.00)	28 (54.90)	
	Male	24 (48.00)	23 (45.10)	
Age [n (%), year]				0.690
	<70	17 (34.00)	20 (39.22)	
	70–79	29 (58.00)	29 (56.86)	
	≥80	4 (8.00)	2 (3.92)	
Height ( $\bar{x}$ ± s, cm)	Average height	165.76 ± 7.80	165.16 ± 6.47	0.635
Weight ( $\bar{x}$ ± s, kg)	Average weight	62.92 ± 8.27	63.64 ± 6.29	0.583
BMI [n (%)]				0.544
	<18.5	7 (14.00)	3 (5.88)	
	18.5–23.9	28 (56.00)	33 (64.71)	
	24–26.9	10 (20.00)	11 (21.57)	
	≥27	5 (10.00)	4 (7.84)	
SBP ( $\bar{x}$ ± s, mmHg)	MSBP	125.80 ± 7.84	123.57 ± 5.49	0.102
DBP ( $\bar{x}$ ± s, mmHg)	MDBP	77.50 ± 8.86	78.53 ± 6.97	0.517
HR ( $\bar{x}$ ± s, bpm)	AvgHR	75.14 ± 7.68	76.55 ± 6.52	0.322
Personal history [n (%)]				
	Smoking	22 (44.00)	21 (41.18)	0.774
	Drinking	18 (36.00)	19 (37.25)	0.896
Disease course ( $\bar{x}$ ± s, year)	Average disease duration	4.04 ± 2.14	3.73 ± 2.10	0.457
NYHA class [n (%)]				0.882
	Ⅰ	0 (0.00)	0 (0.00)	
	Ⅱ	7 (14.00)	6 (11.76)	
	Ⅲ	33 (66.00)	36 (70.59)	
	Ⅳ	10 (20.00)	9 (17.65)	
Medical history [n (%)]				
	Hypertension	30 (60.00)	29 (56.86)	0.749
	Hyperlipidemia	24 (48.00)	25 (49.02)	0.918
	Diabetes	14 (28.00)	16 (31.37)	0.711
	stroke	6 (12.00)	4 (7.84)	0.525

Abbreviation: Mean systolic blood pressure (MSBP), Mean diastolic blood pressure (MDBP), Average heart rate (AvgHR), Body mass index (BMI), New York Heart Association (NYHA).

### Laboratory indicators

Routine laboratory tests (complete blood counts, liver and kidney function tests, and serum electrolytes) showed no significant between-group differences after treatment (Table 2). No clinically significant laboratory abnormalities were observed, and no adverse events were reported in either group.

**TABLE 2:** Routine laboratory test indexes of two groups of patients ( $\bar{x}$ ± s).

Item	Control group (*n* = 50)	QLQX group (*n* = 51)	*t*	*P*
WBC (10^9^/L)				
Before	6.70 ± 1.30	6.68 ± 1.35	0.072	0.942
After	6.77 ± 1.25	6.61 ± 1.39	0.614	0.541
RBC (10^9^/L)				
Before	4.45 ± 0.39	4.57 ± 0.32	−1.698	0.093
After	4.60 ± 0.33	4.54 ± 0.37	0.788	0.433
PLT (10^9^/L)				
Before	214.73 ± 47.52	215.90 ± 46.89	−0.124	0.901
After	215.18 ± 48.48	215.48 ± 45.97	−0.032	0.975
ALT (U/L)				
Before	30.00 ± 8.81	31.25 ± 8.91	−1.698	0.093
After	31.94 ± 8.07	32.77 ± 8.60	−0.500	0.618
AST (U/L)				
Before	30.43 ± 8.86	30.69 ± 8.78	−0.150	0.881
After	31.39 ± 8.42	29.79 ± 9.1	0.915	0.362
BUN (mmol/L)				
Before	5.10 ± 1.12	5.28 ± 0.95	−0.867	0.388
After	5.16 ± 1.08	5.22 ± 1.01	−0.271	0.787
Cre (μmol/L)				
Before	67.67 ± 5.74	67.33 ± 5.24	0.316	0.752
After	68.37 ± 4.97	66.67 ± 5.81	1.570	0.120
K^+^ (mmol/L)				
Before	4.40 ± 0.48	4.49 ± 0.41	−1.039	0.301
After	4.44 ± 0.44	4.45 ± 0.45	−0.147	0.884
NA^+^ (mmol/L)				
Before	138.60 ± 4.35	137.71 ± 3.77	1.088	0.279
After	137.86 ± 3.83	138.41 ± 4.28	−0.677	0.500
CA^2+ ^(mmol/L)				
Before	2.37 ± 0.15	2.39 ± 0.14	0.317	0.752
After	2.39 ± 0.14	2.38 ± 0.15	0.342	0.733

Abbreviation: Before treatment (Before), After treatment (After).

### Echocardiographic parameters

Before treatment, including left ventricular ejection fraction (LVEF), left ventricular end-diastolic diameter (LVEDD), left ventricular posterior wall thickness (LVPWT), interventricular septal thickness (IVS), and left atrial diameter (LAD) were similar between groups. After treatment, there were no statistically significant between-group differences in these echocardiographic parameters (Table 3).

**TABLE 3:** Related indexes of echocardiography before and after treatment in two groups ( $\bar{x}$ ± s).

Item	Control group (*n* = 50)	QLQX group (*n* = 51)	*t*	*P*
LVEF (%)				
Before	43.82 ± 6.92	42.63 ± 5.39	0.967	0.336
After	45.21 ± 6.84	45.41 ± 6.30	0.618	0.538
*t*	−0.779	−1.300		
*P*	0.438	0.196		
LVEDD (mm)				
Before	58.47 ± 8.85	59.01 ± 6.76	−0.344	0.732
After	57.76 ± 9.04	58.67 ± 7.58	−0.548	0.585
*t*	0.398	0.240		
*P*	0.692	0.811		
LVPWT (mm)				
Before	8.70 ± 1.32	8.75 ± 1.48	−0.158	0.875
After	8.68 ± 1.38	8.73 ± 1.52	−0.175	0.861
*t*	0.089	0.058		
*P*	0.929	0.954		
IVS				
Before	9.04 ± 1.38	9.24 ± 1.54	−0.676	0.500
After	8.98 ± 1.35	9.13 ± 1.51	−0.514	0.608
*t*	0.220	0.365		
*P*	0.826	0.716		
LAD (mm)				
Before	44.00 ± 5.22	45.09 ± 6.06	−0.964	0.337
After	44.10 ± 5.33	45.05 ± 5.70	−0.862	0.391
*t*	−0.097	0.032		
*P*	0.923	0.975		

Abbreviation: Before treatment (Before), After treatment (After).

### Heart function classification

After 8 weeks, NYHA class (as defined in Methods) improved in both groups, with greater shift toward lower classes (i.e., better function) in the QLQX group (Table 4). The proportion of patients with moderate-to-severe dysfunction (class III/IV) decreased more in the QLQX group than in the control group. The predefined therapeutic response rate (marked + effective) was higher with QLQX than with control. No participant experienced a deterioration in NYHA class.

**TABLE 4:** Comparison of cardiac function grading after treatment (n [%]).

Item		Control group (*n* = 50)	QLQX group (*n* = 51)	Total (n = 101)	*χ* ^*2*^	*P*
NYHA class	Ⅰ	9 (18.00)	14 (27.45)	23 (22.77)	12.006	<0.01
	Ⅱ	12 (24.00)	24 (47.06)	36 (35.64)		
	Ⅲ	24 (48.00)	9 (17.65)	33 (32.67)		
	Ⅳ	5 (10.00)	4 (7.84)	9 (8.91)		
Efficacy	Ineffective	24 (48.00)	10 (19.61)	34 (33.66)	11.98	<0.01
	Effective	24 (48.00)	31 (60.78)	55 (54.46)		
	Significantly effective	2 (4.00)	10 (19.61)	12 (11.88)		

Abbreviation: New York Heart Association (NYHA).

### Minnesota Living with Heart Failure Questionnaire (MLHFQ) score

**FIGURE 1:**
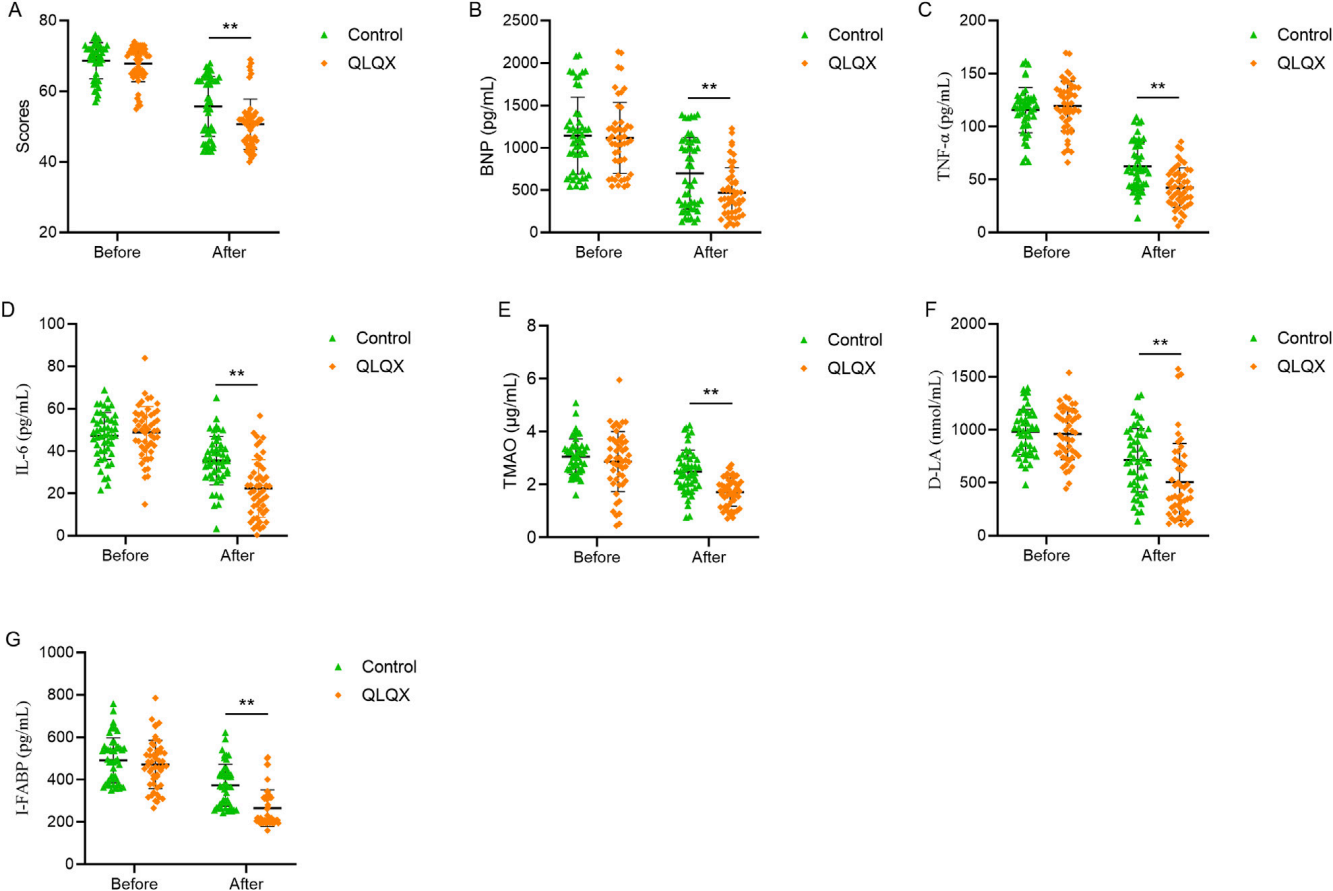
Effects of Qili Qiangxin administration improved clinical and biomarker profile in patients with chronic heart failure. **(A)** Minnesota Living with Heart Failure Questionnaire (MLHFQ) scores. **(B)** Serum B-type natriuretic peptide (BNP) levels. **(C,D)** Circulating pro-inflammatory cytokines TNF-α and IL-6. **(E)** Trimethylamine N-oxide (TMAO), a gut microbiota-derived metabolite. **(F,G)** Intestinal permeability biomarkers D-lactate (D-LA) and intestinal fatty acid-binding protein (I-FABP). All panels compare pre- and post-treatment values between Qili Qiangxin (QLQX) and control groups. **p < 0.01, vs. Control.

MLHFQ scores did not differ between groups at baseline. Scores improved from baseline in both groups, the reduction was significantly larger in the QLQX group (Table 5; Figure 1A), indicating greater improvement in health-related quality of life.

**TABLE 5:** Comparison of Minnesota Living with Heart Failure Questionnaire score after treatment in two groups ( $\bar{x}$ ± s).

Item	Control group (*n* = 50)	QLQX group (*n* = 51)	*t*	*P*
Scores				
Before	68.70 ± 5.11	67.96 ± 5.13	0.725	0.470
After	55.72 ± 8.51	50.55 ± 7.13	3.307	<0.01
*t*	9.244	14.158		
*P*	<0.01	<0.01		

Abbreviation: Before treatment (Before), After treatment (After).

### BNP, TNF-α, IL-6, and TMAO

Baseline blood BNP levels were comparable between groups. BNP decreased significantly in both groups after treatment, with a larger reduction in the QLQX group (Table 6; Figure 1B). Baseline serum TNF-α and IL-6 levels did not differ between groups. Both cytokines decreased significantly after treatment, with greater reductions in the QLQX group (Figures 1C,D). Baseline serum TMAO levels, quantified by liquid chromatography-tandem mass spectrometry, was similar between groups and declined significantly after treatment in both groups, with a larger decrease in the QLQX group (Figure 1E).

**TABLE 6:** The BNP, TNF-α, IL-6, TMAO Levels between the two groups after treatment ( $\bar{x}$ ± s).

Item	Control group (*n* = 50)	QLQX group (*n* = 51)	*t*	*P*
BNP (pg/mL)				
Before	1080.90 ± 353.18	1056.90 ± 315.39	0.360	0.719
After	698.54 ± 422.61	480.53 ± 300.52	2.983	<0.01
*t*	5.093	8.927		
*P*	<0.01	<0.01		
TNF-α (pg/mL)				
Before	115.69 ± 21.38	119.36 ± 23.35	−0.823	0.412
After	62.62 ± 22.48	42.48 ± 18.48	4.925	<0.01
*t*	5.097	6.439		
*p*	<0.01	<0.01		
IL-6 (pg/mL)				
Before	47.21 ± 11.08	48.95 ± 12.10	−0.751	0.454
After	35.48 ± 11.45	22.51 ± 13.47	5.209	<0.01
*t*	5.208	5.427		
*p*	<0.01	<0.01		
TMAO (μg/mL)				
Before	3.06 ± 0.67	2.89 ± 1.13	0.957	0.341
After	2.49 ± 0.81	1.72 ± 0.53	5.675	<0.01
*t*	3.861	6.731		
*P*	<0.01	<0.01		

Abbreviation: Before treatment (Before), After treatment (After).

### Peripheral blood markers of intestinal mucosal permeability

Serum D-LA and I-FABP levels did not differ between groups at baseline. Both markers decreased significantly after treatment, with more pronounced reductions in the QLQX group (Table 7; Figures 1F,G).

**TABLE 7:** Comparison of permeability of peripheral blood intestinal mucosa between the two groups after treatment ( $\bar{x}$ ± s).

Item	Control group (*n* = 50)	QLQX group (*n* = 51)	*t*	*P*
D-LA (nmol/mL)				
Before	980.09 ± 212.91	967.68 ± 244.35	0.272	0.786
After	715.39 ± 298.82	505.39 ± 361.23	3.18	<0.01
*t*	5.101	7.57		
*P*	<0.01	<0.01		
I-FABP (pg/mL)				
Before	491.68 ± 106.65	470.76 ± 113.29	0.955	0.342
After	373.97 ± 99.05	264.69 ± 86.12	5.921	<0.01
*t*	5.719	5.341		
*P*	<0.01	<0.01		

Abbreviation: Before treatment (Before), After treatment (After).

### Baseline correlation analysis

At baseline, TMAO, TNF-α, and IL-6 were positively correlated with BNP (r = 0.875, 0.630, and 0.737, respectively; all p < 0.001) and with MLHFQ scores (r = 0.065, 0.198, and 0.116, respectively; all p < 0.001), and negatively correlated with LVEF (r = −0.472, −0.145, and −0.115, respectively; all p < 0.001) (Table 8). With increasing NYHA class, serum TMAO, TNF-α, IL-6, D-LA, I-FABP, and BNP levels, as well as MLHFQ scores, increased, whereas LVEF decreased (Table 9). Serum D-LA and I-FABP were positively correlated with TMAO (r = 0.671 and 0.580, respectively; both p < 0.001), TNF-α (r = 0.352 and 0.461, respectively; both p < 0.001), and IL-6 (r = 0.484 and 0.470, respectively; both p < 0.001), supporting an association between increased intestinal permeability and systemic inflammation (Table 10).

**TABLE 8:** Correlation of TMAO, TNF-α and IL-6 with MLHFQ score, BNP and LVEF.

Item	MLHFQ score	BNP (pg/mL)	LVEF (%)
TMAO (μg/mL)			
*r*_*s*_	0.065	0.875	−0.472
*P*	<0.001	<0.001	<0.001
TNF-α (pg/mL)			
*r*_*s*_	0.198	0.630	−0.145
*P*	<0.001	<0.001	<0.001
IL-6 (pg/mL)			
*r*_*s*_	0.116	0.737	−0.115
*P*	<0.001	<0.001	<0.001

Abbreviation: Minnesota Living with Heart Failure Questionnaire (MLHFQ).

**TABLE 9:** The association of related detection indexes with different cardiac function grades before treatment ( $\bar{x}$ ± s).

Item	NYHA Ⅱ (n = 13)	NYHA Ⅲ (n = 69)	NYHA Ⅳ (n = 19)	*P*
TMAO (μg/mL)	2.08 ± 0.36	3.08 ± 0.68	3.87 ± 0.37	<0.001
TNF-α (pg/mL)	100.92 ± 21.81	118.08 ± 19.77	130.13 ± 19.75	<0.001
IL-6 (pg/mL)	38.52 ± 7.19	48.67 ± 12.21	55.4 ± 5.91	<0.001
D-LA (nmol/mL)	818.15 ± 133.36	979.41 ± 188.3	1160.9 ± 274.98	<0.001
I-FABP (pg/mL)	410.79 ± 162.27	405.2 ± 170.53	534.56 ± 194.13	<0.001
BNP (pg/mL)	634.63 ± 114.65	1085.16 ± 291.69	1493.6 ± 147.05	<0.001
LVEF (%)	48.4 ± 4.77	43.74 ± 6.72	38.58 ± 4.29	<0.001
MLHFQ score	60.76 ± 3.39	69.28 ± 4.1	73.39 ± 1.21	<0.001

Abbreviation: New York Heart Association (NYHA), Minnesota Living with Heart Failure Questionnaire (MLHFQ).

**TABLE 10:** The correlation between D-LA, I-FABP and TMAO, TNF-α, IL-6 in patients.

Item	TMAO (μg/mL)	TNF-α (pg/mL)	IL-6 (pg/mL)
D-LA (nmol/mL)			
*r*_*s*_	0.671	0.352	0.484
*P*	<0.001	<0.001	<0.001
I-FABP (pg/mL)			
*r*_*s*_	0.580	0.461	0.470
*P*	*<0.001*	*<0.001*	*<0.001*

## Discussion

Classification of chronic heart failure (CHF) by left ventricular ejection fraction (LVEF) remains central to clinical decision-making, while its limitations are well recognized (Triposkiadis et al., 2024). For heart failure with reduced ejection fraction (HFrEF) and mildly reduced ejection fraction (HFmrEF), evidence-based pharmacotherapies—including diuretics, renin–angiotensin system inhibitors, SGLT2 inhibitors, as well as ivabradine or digitalis in selected patients—reduce morbidity and mortality in randomized trials (Kaul, 2025). In contrast, the management of with preserved ejection fraction (HFpEF) remains suboptimal; nearly half of CHF patients fall into this phenotype, yet few therapies have conclusively improved survival (Hamo et al., 2024; Rafei et al., 2025). These gaps support the exploration for adjunctive strategies that act on non-canonical pathways implicated in CHF progression.

In this single-center study, the traditional Chinese medicine formulation Qili Qiangxin (QLQX), used as an adjunct to guideline-directed medical therapy, was associated with improvements in functional status and quality of life, together with favorable changes in biomarkers of systemic inflammation and intestinal barrier integrity. Specifically, compared with controls, patients receiving QLQX experienced greater improvement in New York Heart Association (NYHA) class and lower scores on the Minnesota Living with Heart Failure Questionnaire (MLHFQ). These results are consistent with prior clinical and preclinical evidence suggesting that QLQX exerts cardioprotective effects, in part via modulation of inflammatory pathways (Packer, 2023; Wang et al., 2017; Wang et al., 2023; Sun et al., 2016). We also observed a significant reduction in B-type natriuretic peptide (BNP), a biomarker of ventricular wall stress (Tsutsui et al., 2023). Reductions in N-terminal pro–B-type natriuretic peptide (NT-proBNP) with QLQX have similarly been reported in HFrEF populations (Cheang et al., 2024; Liao et al., 2024).

A key and novel observation is the association between QLQX treatment and lower circulating markers of intestinal epithelial injury and permeability. Serum D-lactate (D-LA) and intestinal fatty acid-binding protein (I-FABP) declined significantly with QLQX, consistent with improved mucosal barrier integrity (Li et al., 2023). Impaired intestinal barrier function is a recognized contributor to systemic inflammation in CHF via translocation of microbial products (Liu et al., 2024; Horowitz et al., 2023). Inflammation contributes to CHF pathophysiology, by promoting cardiomyocyte hypertrophy, interstitial fibrosis, and progressive pump dysfunction (Wenzl et al., 2021; Rocca et al., 2020). In parallel, QLQX was associated with significant reductions in the pro-inflammatory cytokines tumor necrosis factor-α (TNF-α) and interleukin-6 (IL-6). Similar cytokine reductions have been reported in patients with dilated cardiomyopathy and in post–myocardial infarction models treated with QLQX (Liao et al., 2024; Xiao et al., 2009). Prior clinical work also described decreases in soluble growth STimulation expressed gene 2 (sST2), galectin-3, and high-sensitivity C-reactive protein (hs-CRP) in elderly heart failure patients, and in prostaglandin E2 and IL-10 in CHF rats, following QLQX administration (Wang et al., 2023; Xiao et al., 2009). In our cohort, I-FABP and D-LA were positively correlated with TNF-α and IL-6, and these inflammatory markers were inversely correlated with LVEF, aligning with the established link between gut barrier dysfunction, systemic inflammation, and myocardial remodeling in heart failure (Amati et al., 2003; Bro-Jeppesen et al., 2017).

QLQX also lowered plasma trimethylamine-N-oxide (TMAO), a gut microbiota-derived metabolite implicated in adverse cardiovascular outcomes via oxidative stress and inflammasome activation (Li et al., 2022; Dong et al., 2021). The concurrent reductions in TMAO, D-LA, and I-FABP suggest that QLQX may favorably influence both the physical integrity of the intestinal barrier and the metabolic output of the gut microbiota. This constellation of effects differs from standard neurohormonal therapies, which have robust effects on natriuretic peptides but limited impact on gut-specific pathways (Ruiz-Bustillo et al., 2025), and from some microbiome-targeted interventions that modulate TMAO without clear improvement in barrier integrity (Haarhuis et al., 2025). Together with prior work implicating intestinal barrier dysfunction as a driver of inflammation in heart failure and highlights the paucity of therapies that directly address it, these observations support a multifaceted mechanism for QLQX (Guivala et al., 2024; Huang and Liu, 2023; Johnson et al., 2025). Causality and pathway specificity, however, require dedicated mechanistic studies.

Cardiomyocyte apoptosis contributes to progressive ventricular remodeling in CHF. Preclinical studies suggest that QLQX attenuates apoptotic signaling and preserves myocardial structure (Zhao et al., 2019; Lu et al., 2020). Although apoptosis was not directly measured in our cohort, the observed decreases in TNF-α and IL-6—cytokines that can promote apoptosis through intrinsic and extrinsic pathways—are consistent with a less pro-apoptotic milieu (Dhingra et al., 2009; Yndestad et al., 2006; Wang et al., 2015). The reduction in TMAO may also contribute by limiting oxidative stress and inflammasome activation (Wang et al., 2024; Wang et al., 2022). These mechanistic links remain hypothesis-generating.

Collectively, these findings suggest that an adjunctive strategy that simultaneously improves intestinal permeability, modulates gut microbial metabolism, and attenuates systemic inflammation may yield clinically meaningful benefits in CHF. The observed improvements in functional status and natriuretic peptides provide a plausible clinical correlate of these biomarker changes.

This study has several limitations. First, the single-center design with moderate sample size may limit generalizability. Second, although we assessed biomarkers of intestinal permeability and inflammation, we did not perform gut microbiota sequencing or measure circulating lipopolysaccharide, which would provide deeper mechanistic insights translocation and host response. Third, the follow-up period was relatively short; longer-term studies are needed to assess sustainability of benefits and effects on clinical outcomes (hospitalization and mortality). Future research should include larger, multicenter, long-term trials focused on specific HF phenotypes (e.g., HFpEF) and incorporate multi-omics approaches to precisely delineate the molecular targets of QLQX and its interaction with the gut microbiome.

The safety of QLQX has been evaluated through preclinical toxicological studies and post-marketing surveillance in China. As an approved medicinal product, its real-world clinical use supports a favorable benefit–risk profile for the indicated population when used as directed. All botanical drugs are processed in compliance with the Chinese Pharmacopoeia and manufactured under Good Manufacturing Practice to ensure quality and authenticity. The safety profiles of principal marker plant metabolites and the finished product have been characterized by the manufacturer. Nonetheless, potential synergistic or antagonistic interactions among plant metabolites within the formulation warrant continued pharmacovigilance and targeted clinical investigation across diverse patient groups and comorbidities.

## Conclusion

Qili Qiangxin appears to be a promising adjunct to guideline-directed therapy in chronic heart failure. In this study, QLQX was associated with improved cardiac functional status and quality of life, lower natriuretic peptides, and reductions in biomarkers of intestinal epithelial injury, gut-derived metabolism (TMAO), and systemic inflammation. By concurrently modulating dysregulated inflammatory signaling and impaired intestinal barrier integrity—two interrelated domains implicated in CHF progression—QLQX may complement standard therapies. These findings support adjunctive use; efficacy as monotherapy is not established. Large-scale, multicenter, and longer-duration trials with mechanistic endpoints are needed to validate these observations, clarify causal pathways, and define the optimal role of QLQX in CHF care.

## Data Availability

The original contributions presented in the study are included in the article/Supplementary Material, further inquiries can be directed to the corresponding authors.
