# Supplementary material for: Qili Qiangxin ameliorates chronic heart failure: a randomized clinical trial of biomarkers, inflammation, and cardiac outcomes
Source: Front Pharmacol. 2025 Sep 30;16:1605944. doi: 10.3389/fphar.2025.1605944 (PMC12518405; doi:10.3389/fphar.2025.1605944)
Supplement: Supplementary file 1 [file Table1.docx]

Table S1 Botanical composition of Qili Qiangxin for 100 capsules

| Botanical Name (Family) | Accepted Pharmacopeial Drug Name | Quantity (g) |
| --- | --- | --- |
| Astragalus mongholicus Bunge [Fabaceae] | Astragali Radix | 45 |
| Panax ginseng C.A.Mey. [Araliaceae] | Ginseng Radix et Rhizoma | 22.5 |
| Salvia miltiorrhiza Bunge [Lamiaceae] | Salviae miltiorrhizae Radix et Rhizoma | 22.5 |
| Alisma orientale (Sam.) Juz. [Alismataceae] | Alismatis Rhizoma | 22.5 |
| Aconitum carmichaelii Debx. [Ranunculaceae] | Aconiti Lateralis Radix Praeparata | 11.25 |
| Descurainia sophia (L.) Webb ex Prantl [Brassicaceae] | Descurainiae Semen | 15 |
| Periploca sepium Bunge [Apocynaceae] | Periplocae Cortex | 18 |
| Cinnamomum cassia (L.) J.Presl [Lauraceae] | Cinnamomi Ramulus | 9 |
| Carthamus tinctorius L. [Asteraceae] | Carthami Flos | 9 |
| Polygonatum odoratum (Mill.) Druce [Asparagaceae] | Polygonati Odorati Rhizoma | 7.5 |
| Citrus reticulata Blanco [Rutaceae] | Citri Reticulatae Pericarpium | 7.5 |
